# Development of a Web-Based Mindfulness Program for People With Multiple Sclerosis: Qualitative Co-Design Study

**DOI:** 10.2196/19309

**Published:** 2021-03-02

**Authors:** Amy-Lee Sesel, Louise Sharpe, Heidi N Beadnall, Michael H Barnett, Marianna Szabo, Sharon L Naismith

**Affiliations:** 1 School of Psychology University of Sydney Sydney Australia; 2 Brain and Mind Centre University of Sydney Sydney Australia; 3 Neurology Department Royal Prince Alfred Hospital Sydney Australia

**Keywords:** multiple sclerosis, mindfulness, depression, program development, internet intervention, qualitative research

## Abstract

**Background:**

Mindfulness-based stress reduction is an efficacious treatment for people with chronic health problems; however, it is highly intensive and time-consuming, which is a barrier for service provision.

**Objective:**

This study aims to develop an internet-delivered adapted version of mindfulness-based stress reduction for people with multiple sclerosis to make the intervention more accessible.

**Methods:**

We co-designed a web-based mindfulness program with end users, that is, people with multiple sclerosis (N=19). Iterative feedback was also collected from a subsample of the initial group of end users (n=11), and the program was reviewed by experts (n=8).

**Results:**

We identified three main themes common to people with multiple sclerosis: dealing with uncertainty and fears for the future, grief and loss, and social isolation. These themes were incorporated into narratives throughout the program. People with multiple sclerosis who reviewed the program gave feedback that the program was relatable, feasible, and acceptable. Experts agreed that the program appropriately represented the main tenets of mindfulness. Iterative feedback was used to further refine the program.

**Conclusions:**

The web-based mindfulness program that we developed was viewed positively by both experts and end users. The program reflects common concerns for people with multiple sclerosis and has the potential to meet important unmet psychological needs. A randomized controlled trial was planned to determine the efficacy of the program.

## Introduction

### Background

People with multiple sclerosis live with high rates of depression and anxiety, with prevalence rates of approximately 31% [1] and 22% [1,2], respectively. However, there is relatively little literature on psychological treatments for people with multiple sclerosis compared with such treatments for people with other chronic health conditions, and the results of these trials are mixed [3-7].

A recent meta-analysis of 13 studies in multiple sclerosis (MS) [8] revealed that psychosocial interventions significantly improve depression, anxiety, fatigue, and mental and total health-related quality of life (HRQoL). However, effect sizes were small, and for physical HRQoL, the effect was moderated by therapy type. Specifically, although there was no clear benefit of cognitive behavior therapy (CBT) for physical HRQoL, other psychosocial therapies such as relaxation and mindfulness had a moderate effect (*d*=0.570).

A single study of mindfulness-based stress reduction (MBSR) [9] demonstrated the largest effect sizes of all included studies for depression (*d*=0.8) and anxiety (*d*=0.6). This was a high-quality randomized controlled trial (RCT) of 150 people with multiple sclerosis, showing that face-to-face MBSR was effective across a range of outcomes compared with treatment as usual. Indeed, mindfulness has been shown to have significant mental health benefits for people with a range of physical health conditions, including chronic pain, fibromyalgia, arthritis, and cancer [10,11]. However, the number of trials in the MS literature is limited, and the sample sizes are generally small [3-7]. There is another more recent study of 62 people with multiple sclerosis. This study did not find any significant differences between MBSR and an active control group. However, this study was underpowered [12].

Both RCTs in MS were based on the traditional MBSR program [13] and involved a high dosage of face-to-face intervention (27 hours and 22 hours, respectively), including a full-day workshop. Such intensive treatments are costly, and barriers such as reduced mobility limit accessibility for people with multiple sclerosis. Finding alternative modes of delivery could potentially improve the access, cost-effectiveness, and scalability of interventions. Internet-delivered mindfulness programs have been found to be effective in mental health settings [14] and in chronic disease populations such as cancer, irritable bowel syndrome, and fibromyalgia [15-17]. There is only 1 RCT evaluating the efficacy of online meditation training for people with multiple sclerosis. This study found significant treatment effects for HRQoL, depression, anxiety, and sleep problems at postintervention, but the benefits were not maintained [18]. Although this program was an MS-specific intervention, it was not evident that people with multiple sclerosis were involved in its design. Further research in this field is needed to clarify the mixed results of web-based mindfulness studies and to determine whether a more tailored intervention with consumer input would lead to sustained, longer-term improvements in mental health.

### Objectives

The aim of this study is to develop a web-based mindfulness program tailored specifically for people with multiple sclerosis via a qualitative investigation of (1) the psychological experiences and unmet needs of people with multiple sclerosis, (2) attitudes toward a proposed web-based mindfulness program designed to address such needs, and (3) iterative feedback from people with multiple sclerosis and experts in the field.

## Methods

### Recruitment

Participants were recruited from the MS Clinic, Brain and Mind Centre, Sydney, Australia, between August 2017 and July 2018. Ethics approval was obtained from the University of Sydney Human Research Ethics Committee (2016/049). Potential participants were approached at the MS Clinic waiting room consecutively and given an invitation letter with the opportunity to fill out their contact details. We did not exclude participants with comorbid physical or mental health conditions. Participants were required to be older than 18 years and to have a neurologist-confirmed diagnosis of MS. Interview times were arranged via telephone and were conducted face-to-face at the MS Clinic at an appointed time, with written consent, by a registered psychologist (AS).

### Data Collection

Demographic data were collected via interviews and self-report questionnaires. To characterize the sample, we administered valid and reliable measures of depression, anxiety, and pain. Depression symptomatology was measured using the Center for Epidemiological Studies for Depression questionnaire [19] (CES-D; 20-item; range 0-60); anxiety was measured using the State-Trait Anxiety Inventory [20] (STAI) via 2 subscales, assessing state and trait anxiety (both 20-item; range 20-80); and pain was measured using a 10-point visual analogue scale [21]. Semistructured interviews (approximately 50 min in length on average) were recorded and transcribed verbatim. All participants were asked the same semistructured interview questions (Multimedia Appendix 1) with appropriate follow-up questions.

### Data Analysis

Data were analyzed using NVivo Qualitative Data Analysis Software [22], via inductive coding at the semantic level, consistent with the Miles and Huberman framework for qualitative data analysis [23]. Analyses were ceased at the point of theme saturation, where 3 transcripts revealed no new themes. Individual transcripts were analyzed and coded independently by 2 authors (AS and LS). The researchers then met with a third researcher (SN) to confirm the themes and coding framework. Any disagreements were resolved by consensus.

### Program Development

The web-based mindfulness program was developed on the basis of core components from the MBSR program by Kabat-Zinn [24], with the exception of yoga training. Hatha yoga was omitted from the program because it could not be sufficiently supervised and because the program was designed to target people with multiple sclerosis with varying degrees of mobility. The program was tailored to address the main themes derived from the qualitative interviews with people with MS by integrating case examples from unique, fictional characters that varied in terms of age, gender, race, disease course, and duration. These case examples were used to normalize the day-to-day challenges of people with multiple sclerosis and demonstrate how MBSR could be used to manage MS-related symptoms and improve HRQoL. The program comprised five 15-min web-based modules (Table 1), designed to be delivered over 8 weeks. We opted for 5 modules because there is some evidence that having at least four modules is optimal in other internet interventions (eg, CBT insomnia) [25], but we were unable to provide all meditations in the 4 modules. Furthermore, there is evidence of good adherence to other CBT-based interventions that offer 5 modules over 8 weeks [26,27].

**Table 1:** Web-based mindfulness program content.

Module	Topics covered	Mindfulness-based stress reduction principles	Formal practice	Informal practice
Module 1: Introduction to mindfulness meditation	Anxiety	Definition of mindfulness^a^ Benefits of mindfulness Simple awareness^b^	Awareness of breath meditation	Choose one daily activity to do mindfully
Module 2: Recognizing signs of stress	Stress and fatigue	Definition of stress Signs of stress The body scan^c^	Body scan meditation	Practice brief body scan before a stressful event
Module 3: Dealing with difficult sensations and emotions	Pain	Cultivating equanimity^d^ Swapping judgment for curiosity Letting go	Sitting meditation (open awareness)	Cultivate equanimity toward an everyday conflict
Module 4: Dealing with difficult thoughts	Low mood	Using the breath as an anchor The 4-step process of nonidentification^e^ The mountain meditation	Mountain meditation	Write down a thought, practice nonidentification, and then write it down again
Module 5: Mindful communication, self-compassion, and relapse prevention	Social isolation	Active listening and responding (vs reacting) Cultivating self-compassion Recap of mindfulness program	Loving-kindness meditation	Complete mindful listening exercise with a partner

^a^Mindfulness: the awareness that arises when we pay attention on purpose, in the present, nonjudgmentally.

^b^Simple awareness: observing what is happening by focusing your attention completely on one thing at a time, without judging your experience in any way.

^c^Body scan: a type of mindfulness meditation that involves concentrating one’s attention on one’s body, moving from the tip of the toes to the top of the head in a systematic, mindful way.

^d^Equanimity: a neutral state of mind toward all experiences, pleasant and unpleasant, regardless of whether they bring pleasure, joy, or misery.

^e^Nonidentification: a technique that involves being conscious of not becoming attached to whatever thoughts arise within your internal experience, taking a step back, and viewing each thought as an impersonal mental event.

The 5 modules were scheduled as follows: module 1 at the start of week 1 and module 2 at the start of week 2. The remaining modules contain instructions for the next 2 weeks, that is, module 3 would be delivered at the start of week 3 and again for the option of repetition, at the beginning of week 4, etc. The amount of time (ie, 1 or 2 weeks) dedicated to each module was decided based on the complexity of the mindfulness concepts and strategies described. It was written by a registered psychologist (AS) with clinical psychology and mindfulness training in collaboration with a clinical psychologist with experience in the development and evaluation of evidence-based psychological interventions for people with chronic health conditions (LS) and a clinical neuropsychologist with experience in the delivery of web-based therapeutic interventions (SN).

Direct, face-to-face feedback on the program was provided by a subsample of the original participants interviewed (n=11), who indicated that they were interested and willing to review the modules of the program. In total, 8 women and 3 men with MS (10/11 relapsing-remitting; age range: 23-63 years) took part in the review process. Two neurologists and 4 mindfulness experts including 2 clinical psychologists, 1 emergency care physician and 1 general practitioner provided written feedback on the program. Feedback was then coded and analyzed inductively, forming subheadings with overarching themes. Feedback from both people with multiple sclerosis and experts was discussed in team meetings among the 3 lead authors (AS, LS, and SN). Further iterations of the program were cocreated where there was consensus and scope to make the suggested changes.

## Results

### Demographics

All participants had a neurologist-confirmed diagnosis of MS (N=19). There were 6 males and 13 females. A total of 63% (12/19) of participants had clinically significant depressive symptoms according to a cut-off score of 16 on the CES-D [28]. Moreover, 47% (9/19) of participants had clinically significant levels of state anxiety, according to a cut-off score of 41, and 42% (8/19) of participants had clinically significant levels of trait anxiety, according to a cut-off score of 44 on the STAI [29]. Descriptive statistics are presented in Table 2.

**Table 2:** Participant demographics (N=19).

Characteristics		Values
Females, n (%)		13 (68)
Age (years), mean (SD)		40.42 (16)
**Employment status, n (%)**		
	Working full-time or part-time	8 (42)
	Unemployed	6 (32)
	Retired	1 (5)
	University student	3 (16)
	High school student	1 (5)
**Type of MS^a^, n (%)**		
	Primary progressive MS	5 (26)
	Relapsing-remitting MS	14 (74)
**Walking ability, n (%)**		
	Wheelchair or scooter	3 (16)
	Ambulatory	16 (84)
**Psychosocial descriptors**		
	Pain now, n (%)	8 (42)
	Pain, mean (SD)^b^	4.48 (4)
	Depressive symptoms, mean (SD)	19.89 (13)
	State anxiety, mean (SD)	39.16 (15)
	Trait anxiety, mean (SD)	43.89 (14)

^a^MS: multiple sclerosis.

^b^On the basis of the 8 participants who reported experiencing pain.

### Thematic Analysis

The thematic analysis of this qualitative study was divided into 3 parts, in accordance with the research aims. For detailed thematic maps, including illustrative example quotations, please see Tables 3-5.

**Table 3:** Part 1: Psychological experiences and unmet needs of people with multiple sclerosis.

Overarching theme and subtheme		Participant quote
**Uncertainty**		
	When another relapse will occur	“...you really don’t know when you’re going to have another episode...it’s a pretty stressful thing.” (P6)
	Fear of progression	“I guess I don’t want to face the fact that yeah, something could happen and when’s it going to happen? I don’t know. Nobody’s knows so I guess.” (P17)
	Potential burden on family	“I suppose my biggest worry at the moment is, how long will I stay, or how could I possibly get a little bit better so I can get more strength so I can actually um, perform better in the workplace...cause my wife would be never the person who could go out and work...I don’t know what would happen to her.” (P1)
	Neurologist appointments	“Anytime I have to come to talk to Dr [name] about something or get results...it just destroys me for a week or two.” (P2)
	Gaps in medical knowledge	“I remember when I first got diagnosed and I asked, what causes this, they said...its multifactorial, which means we don’t know...and you get that answer a lot.” (P8)
	What is MS^a^-related	“Sometimes when I’m fatigued, or having low mood, it’s hard to know whether that’s just a normal thing, or whether it is MS influenced.” (P6)
**Grief and loss**		
	Loss of future plans	“I’m just feeling really heartbroken about having MS...Our whole future and life just completely changed.” (P2)
	Difficulty with acceptance	“There’s an element of why me, poor me...why is this happening, this can’t be happening...and then cry a lot of tears.” (P3)
	Loss of independence	“I can’t do anything that I used to be able to do...I can’t play netball, I can’t play touch footy, I can’t walk in the dark...you lose everything...” (P4)
	Loss of cognitive abilities	“...I’ve missed out on a lot of things in life that you would normally take for granted...like as your young children grow up...I don’t remember those years of them...” (P1)
	Loss of confidence	“I don’t feel like I can rely on my own devices...I wouldn’t have the confidence to go and do something on my own that’s out of the norm, out of my comfort zone, so that’s my thing, is I just feel vulnerable.” (P18)
	Loss of sense of self and identity	“I can’t think normally anymore, and I’m not as quick as I used to be either. Everything’s slowly deteriorating, including me and my thoughts.” (P7)
**Social isolation**		
	Emotional avoidance	“Well, it’s mainly my family, who will sometimes bring it up in conversations...but yeah I really avoid it...” (P3)
	Inappropriate responses of family or friends	“They try to help, but like I’ve had people burst into tears when I tell them, just like really inappropriate because they don’t know how to handle it...” (P2)
	Lack of understanding	“Nobody really understands, and I don’t really have anybody to talk to about it.” (P17)
	Apathy from others	“I’ve been brave enough to step out there and say this is what’s going on for me, and kind of reaching out a hand for some kind of support or acknowledgement, and getting nothing...” (P15)
	Rejection due to stigma	“Yeah, [friends] walked away. Cause I got MS you know...they could catch it!” (P4)
**Availability of support**		
	Lack of follow-up	“Well, I suppose for someone like me I would’ve appreciated some sort of supportive type of stuff, but there was nothing.” (P13)
	Lack of funding or resources	“From my experience with the MS Society, every time I ever went to them, they always said they ran out of money and couldn’t help me.” (P1)
	Not knowing how to access support	“...I didn’t seek out specific psychological treatment for MS...but I don’t know where I would have gone if I did want that.” (P8)
	Lack of therapists with MS knowledge	“I haven’t found anybody [therapist] who specializes in MS.” (P2)
**Group-based support**		
	Avoiding others with more progressive disability	“...if I see somebody very disabled by their MS, it’s really confronting and upsetting to me, because well, I’m lucky that it’s not me now, but there’s no way of telling what it will be like in the future, and that’s really scary.” (P14)
	Difficulties with relatability	“I don’t really want to talk to people my mother’s age with MS.” (P19)

^a^MS: multiple sclerosis.

**Table 4:** Part 2: Attitudes toward the web-based mindfulness program.

Overarching theme and subtheme		Participant quote
**Seeking support online**		
	Anonymity	“I’d probably feel ok because I can do it in the privacy of my own space so it wouldn’t draw attention to me.” (P3)
	Flexibility	“...when I am overtired, when I am hot, when there are issues, I do need that flexibility.” (P14)
	No need for travel	“I don’t think they [online therapies] would replace in-person, but I think they could augment, or be useful in between. And maybe some people would find it really useful if they don’t have access to something local...” (P2)
	Internet security	“I have big phobias about Internet security and things like that.” (P4)
	Vision problems	“I can’t see...” (P5)
**Mindfulness meditation and barriers to practice**		
	Potential efficacy	“I do have an understanding and I do believe it works...it’s very helpful to bring you back into reality, to stop your mind going crazy.” (P3)
	Difficulties with finding time	“I have had trouble making sure I do it every day, like, finding that time and being strict with myself.” (P17)
	Difficulties concentrating	“I get distracted really easily...” (P14)
	Low mood	“I think it’s a depression. And I know if I were to do those things [to improve my mental health] the depression would get better.” (P2)
	Lack of quiet space	“If I go in my room and close the door, my nieces would just run up and start banging on the door shouting ‘Uncle, uncle, uncle’.” (P7)
**Anticipated program use, preferences, and suggestions**		
	Interest in participating	“I like the idea of the program, and I think there are lots of people who it would really help.” (P6)
	Length of meditation	“I would think minimum 20 minutes because I think anything less than that you haven’t really got into it, to me, takes a while to get into the zone.” (P11)
	Case examples	“...if I had a case to go by, I could say yeah ok that sounds familiar, and then go from there...because with a lot of the stuff it’s like is this supposed to happen? Does it happen to people with MS^a^ or does it happen to everyone? So it would be good to have case studies.” (P3)
	Email reminders	“I’d be more likely to actually use the reminder if it was e-mail, because then I can sort of flag it and keep track of it.” (P10)
	Weekly telephone calls	“To me, I would like follow-up contact. Especially if you develop a rapport with somebody, it’s good to have follow-up contact. That definitely helps with learning a concept, to get a certain amount of rapport and follow-up.” (P1)
	More likely to participate if recommended	“I think it would make it less likely for me to get benefit from it because I’d be so skeptical. If one of my doctors said, or if my psychiatrist who had MS said, [to do it] I certainly would.” (P8)
	More likely to participate if tailored to MS	“...if it’s something like ‘Yeah we can help to work on that anxiety, or work on some things’ and its geared towards people with MS, I think that would be really helpful.” (P2)

^a^MS: multiple sclerosis.

**Table 5:** Part 3: Iterative feedback from people with multiple sclerosis and experts in the field.

Overarching theme and subtheme		Participant quote
**Relatability**		
	Relevance of case examples	“Was it from these interviews that you got the stories for your modules and helped plan the program? I ask this as the stories seem very true to life indeed.” (Exp1)^a^
	Identification with program characters	“I think it’s great. I can relate to Jen and see myself embedded in these slides.” (P11)
	Language: too simplistic?	“My impression was that it was speaking to children in terms of the graphics and language like reading them a storybook. Is that what you intended?” (Exp2)^b^
	Language: is acceptable	“I personally have no issue with it. I feel like that's just kind of the 'mode' you're working in.” (P8)
	Graphics: too childish?	“Yes, I also thought the images looked childish.” (P2)
	Graphics: are acceptable	“I personally really like it, because I think the cartoon-style characters get you completely away from stereotypes and to focus on the messages and the content.” (P15)
**Acceptability of program content**		
	Analogies	“Yeah, really good, I think the analogies are good, you know, the monkey chatter in the mind.” (P17)
	Mindfulness principles	“I love the content, the flow and the style.” (P15)
	Mindfulness training	“I think the text that will be spoken is pretty good and I think follows most mainstream advice about mindfulness and how to teach it.” (Exp3)^c^
	Include expert	“Having a mindfulness expert as a supervisor is important.” (Exp4)^d^
	Include scientific evidence	“… the presentation of some of the scientific evidence underpinning mindfulness generally improves acceptance.” (Exp3)

^a^Exp1: Expert 1, neurologist, specialty in multiple sclerosis

^b^Exp2: Expert 2, general practitioner with expertise in the delivery of web-based mindfulness interventions

^c^Exp 3: Expert 3, emergency physician with expertise teaching mindfulness to people with multiple sclerosis

^d^Exp 4: Expert 4, clinical psychologist with mindfulness teacher training.

#### Part 1: The Psychological Experiences and Unmet Needs of People With Multiple Sclerosis

##### Uncertainty

Most participants reported that their psychological experiences were characterized by uncertainty, worry about the future and possible disease progression, and difficulty grappling with unknowns surrounding the disease. The inability to predict possible relapse was a major source of continual stress and anxiety, and it interfered with planning, for example, whether and when they should have children, how long their physical capabilities would allow them to stay in the workforce, and the potential financial burden that they would place on their families. Going to neurologist appointments and having to face the possibility of receiving bad news, such as the identification of new lesions in the brain or spinal cord, or changing treatments, was a source of excessive worry and distress. Some participants worried about whether their physical or cognitive problems were part of their MS or due to other factors, such as the natural process of aging.

##### Grief and Loss

An overarching theme was the participants’ experience of grief and loss due to the gradual decline in their physical and cognitive abilities and their sense of independence and identity. They also expressed anguish and sorrow over the loss of their illness-free life. Many people with multiple sclerosis reported that they were faced with having to give up on future plans and accept changes to their ability to travel and participate in family life. This process of adjustment experienced by people with multiple sclerosis was depicted as challenging and repetitive, as people developed new or worsening symptoms over time. People with multiple sclerosis experiencing cognitive difficulties described experiences of memory loss and how this impacted their daily functioning as well as their ability to recall cherished memories (eg, of their children growing up), which reinforced feelings of disconnection from family life. Those experiencing physical challenges, such as ataxia, muscle weakness, and bladder dysfunction, reported feelings of helplessness and a great sense of vulnerability, as they gradually lost confidence in themselves. Participants across all ages, genders, and types of MS emphasized the need to separate themselves from the disease, to try to lead a *normal life* and preserve their sense of self and identity, which many feared was at risk of erosion.

##### Social Isolation

Participants reported experiencing social isolation and difficulties communicating with people in their support networks about their MS. Barriers to receiving social support included difficulties with participating in social activities due to loss of function and emotional avoidance when faced with opening up to people about their MS as well as other people’s failure to respond appropriately. Some participants reported that they were given unsolicited and often ill-informed advice from friends and family, which was perceived as highly distressing and alienating. Many participants reflected a sense of apathy and disinterest on behalf of their wider social circles and work environment and others’ general lack of understanding that MS is largely an invisible disease. Two participants reported facing blatant rejection from others because of stigma and unfounded beliefs about MS being contagious.

##### Availability of Psychological Support

Most participants reported an unmet need for psychological support, which they attributed to a lack of follow-up, lack of funding or resources, lack of knowledge regarding where to go for psychological support, and lack of psychologists or therapists specializing in MS. Of the 63% (12/19) participants with clinically significant symptoms of depression, only half reported that they were either currently seeing a psychologist or had seen one in the past.

##### Group-Based Psychosocial Support

A major barrier to receiving psychological support was that the overwhelming majority of participants did not want to meet other people with multiple sclerosis in a face-to-face therapeutic setting, despite wanting to know how others cope. This was largely because meeting people with more severe symptomatology or disability generated feelings of uncertainty and fear around their own illness progression. For representative participant quotations for each subtheme, please see Table 3.

These main themes underlying the psychological experiences of people with multiple sclerosis were incorporated into the program development through the use of fictional characters featured in each module, whose stories and case examples reflected the common challenges, and mental-health related difficulties that participants reportedly faced. For screenshot examples, please see Multimedia Appendix 2.

#### Part 2: Attitudes Toward a Proposed Web-Based Mindfulness-Based Program

##### Attitudes Toward Internet Use

Participants reported positive attitudes toward seeking psychological support online. It is widely regarded as convenient, easy, and allowing access to therapeutic support with a sense of anonymity, in the privacy and comfort of their homes, without the need for travel. Reservations toward the use of an internet-delivered psychological intervention were noted by 11% (2/19) participants. One experienced MS-related vision problems and another expressed apprehension surrounding internet security.

##### Attitudes Toward Mindfulness

Participants’ experiences and knowledge of mindfulness varied from having no understanding of what it was to having years of experience. Potential barriers to participating in the proposed mindfulness program included not having enough time in the day to meditate, difficulties getting in a routine, problems with attention and concentration, low mood, and inability to find a quiet space to meditate, particularly for those with young families.

##### Anticipated Use, Program Preferences, and Suggestions

Most participants reported that they would be interested in participating in the proposed web-based mindfulness intervention (see *Methods* section). People with multiple sclerosis with more experience with mindfulness meditation tended to advocate for longer meditation practices. Features that were identified to engage participants in the program and reduce dropout included incorporating case examples, email reminders, and guiding participants through the program via weekly telephone calls. Three participants reported that they would be more likely to participate in the program if their neurologist or psychiatrist recommended it. Many reported that a program tailored to people with multiple sclerosis was preferable compared with a generic web-based mindfulness program. For representative participant quotations, please see Table 4.

#### Part 3: Iterative Feedback From People With Multiple Sclerosis and Experts in the Field

##### Relatability of the Program

Most people with multiple sclerosis found the program highly relatable to their experiences of living with MS. In total, 5 participants and 1 neurologist gave specific positive feedback about the case examples. Reservations about the relatability of the program were expressed by 1 expert and 1 participant (who held a PhD) who commented that the program was too simplistic and could be perceived as patronizing. Following this feedback, participants and experts were explicitly asked about their views in this regard, and these concerns were not widely shared by other respondents. Some changes to the program graphics and language were incorporated to further improve the relatability of the program: graphics were changed to make the characters look older and the scenes more realistic, and some of the language was edited to be less repetitive and more direct.

##### Acceptability of Program Content

In terms of the program content, 8 participants gave specific, positive feedback on the analogies used to explain mindfulness concepts (eg, *monkey chatter*) and how the mindfulness concepts were presented. Experts in mindfulness commented that the concepts were well explained and consistent with MBSR principles; however, some suggested involving a mindfulness expert, which we did. One area in which there was disagreement among experts was on the degree to which more reference to research should be included. Some experts recommended integrating scientific evidence on the efficacy of mindfulness into the program to improve treatment adherence, whereas others disagreed. We decided that including specific scientific research and results of studies was not necessary. For representative quotations of the feedback provided by participants and experts in the field, please see Table 5.

## Discussion

### Principal Findings

Participants reported psychological support as a common unmet need and were generally positive about the idea of an MS-specific, web-based mindfulness intervention. Among the participants, the anticipated use of such a program was high, particularly if it was endorsed by treating physicians. A thematic analysis of 19 face-to-face interviews with people with multiple sclerosis revealed 3 overarching themes characterizing their psychological experiences: uncertainty, grief and loss, and social isolation. These themes were used in the development of narratives throughout the mindfulness program.

The first theme of uncertainty is unsurprising, considering that MS is an unpredictable illness. Participants indicated that they were fearful of progression, worried about whether symptoms might indicate deterioration, and worried about relapse. The lack of knowledge about the causes of MS exacerbated the uncertainty and worry and made attending medical appointments the source of anxiety. For some, the fear of progression was related to fear of becoming a burden on their family. Fears of progression have been previously documented in MS and found to be associated with poorer psychosocial outcomes [30]; therefore, we featured these throughout the program.

The second theme was grief and loss. Participants described the struggle to accept the limitations imposed on them by MS. They experienced losses of their future plans, which led to a loss of independence. Specific losses such as loss of cognitive abilities and loss of confidence led to a loss of a sense of self and identity. The importance of loss and its impact on self and identity have been previously described as important in the context of adjustment to illness [31]. Hence, we represented these struggles in the narratives developed for the program.

The third theme was social isolation. In terms of social isolation, people with MS felt that family and friends could not understand their difficulties, which sometimes led to inappropriate responses or apathy from others. Some people with MS described having lost friendships, which they experienced as rejection due to the stigma of having MS. Many people with MS described emotional avoidance, which allowed them to not express their emotions to others as a way of trying to avoid negative responses from others. However, this led to an overwhelming feeling of social isolation, which has been previously recognized in the literature [32].

We used these qualitative results to provide narratives to supplement the mindfulness content of our program. Once a draft version of the program was available on the basis of early interviews, we also gained feedback from both the people with multiple sclerosis and experts in MS and mindfulness. Iterative feedback allowed modifications to be made to the program content and graphics to improve relatability and ensure adherence to MBSR principles. However, overall feedback from mindfulness experts, neurologists, and people with multiple sclerosis were positive. The adapted mindfulness program appeared to be an acceptable and highly relatable program, tailored to the unmet needs and experiences of people with multiple sclerosis, and consistent with the basic principles and teachings of MBSR by Kabat Zinn [24].

As previously described, the primary reason for including the psychological experiences of people with multiple sclerosis was to ensure that the narratives included in our program were relevant to the end users of the program. It seems that the issues raised in our sample were similar to those previously raised in the literature. Prior research has shown that there is a lack of psychological support and continuity of psychosocial care after receiving the initial diagnosis [33,34]. Previous studies have highlighted the difficulties in accepting uncertainty surrounding the diagnosis, treatment, and prognosis of MS [35,36]. Indeed, *illness uncertainty* has been found to predict adjustment problems for people with multiple sclerosis, over and above demographic and disease characteristics [37], and the experience of fears of relapse or progression are well documented. Experiences of grief and loss [38] as well as difficulties in maintaining one’s sense of self in the face of physical and cognitive decline have been reported in the literature [31,39,40]. Furthermore, beliefs of people with multiple sclerosis about their illness and illness identity have been found to play a significant role in their psychological adjustment [41]. Previous research suggests that stereotypical images of a person in a wheelchair are regarded as negative symbols of loss, disability, and death for people with multiple sclerosis [42]. This may provide a possible explanation as to why the majority of participants in this study expressed disinterest in participating in group-based psychological interventions or support groups, as participants said they particularly wanted to avoid seeing people with more severe disease than themselves. Finally, it is well known that the psychological well-being of people with multiple sclerosis is worsened by social isolation and difficulties in communicating effectively about the disease with family and friends. In many cases, these feelings of abandonment, isolation, and social withdrawal are reported years after the initial diagnosis [40,43,44]. Commonalities between the themes identified from the people with multiple sclerosis in our study and those reported in the literature indicate that this innovative web-based mindfulness program is likely to be relatable to the target population.

Given the difficulties with anxiety and uncertainty about the future and the experiences of grief and loss experienced by people with multiple sclerosis, a mindfulness-based approach that focuses on the *present moment*, cultivating *acceptance* and *letting go,* seems an appropriate intervention for reducing psychological distress and improving HRQoL. Furthermore, the delivery of a mindfulness intervention, tailored to the needs and experiences of people with multiple sclerosis via the web, can be used to normalize the challenges of living with the disease and decrease feelings of isolation while maintaining anonymity and increasing access to psychological support.

### Limitations

Although we attempted to interview a range of people with multiple sclerosis with various demographic and disease characteristics, we acknowledge that the experiences of people with multiple sclerosis reported here were a reflection of a small sample from one recruitment center. Another limitation of this study was that the reviewers were shown PowerPoint versions of the modules and not the web-based multimedia versions, which would have allowed for further analysis of the program’s usability. Finally, the evaluation of efficacy was beyond the scope of this study. A study protocol for an RCT evaluating the efficacy of the mindfulness program described in this study has been developed [45]. Clearly, the results of an adequately powered RCT are needed to determine the efficacy of the program.

### Conclusions

In summary, we have developed a web-based mindfulness intervention using an iterative co-design process that reflects the common challenges reported by people with multiple sclerosis. We included narratives that were developed from the themes derived from a qualitative study. The modules appropriately reflected the principles of MBSR, and the included narratives were viewed as relatable by people with multiple sclerosis. Future research will determine whether the developed intervention is effective. If it proves to be beneficial, the developed program would likely meet an important need for people with multiple sclerosis.

## Appendix

Multimedia Appendix 1 Interview questions.

Multimedia Appendix 2 Screenshots from the program, as shown to participants.

## Abbreviations

CBT: cognitive behavior therapy
CES-D: Center for Epidemiological Studies for Depression questionnaire
HRQoL: health-related quality of life
MBSR: mindfulness-based stress reduction
MS: multiple sclerosis
RCT: randomized controlled trial
STAI: State-Trait Anxiety Inventory
