# Supplementary material for: Development of a Web-Based Mindfulness Program for People With Multiple Sclerosis: Qualitative Co-Design Study
Source: J Med Internet Res. 2021 Mar 2;23(3):e19309. doi: 10.2196/19309 (PMC7967236; doi:10.2196/19309)
Supplement: Multimedia Appendix 1 [file jmir_v23i3e19309_app1.docx]

**Interview Questions:**

**Current Psychological Difficulties/ Issues Associated with Diagnosis of MS**

- Do you think there is adequate psychological support provided for people with MS that specifically addresses the issues that you face?
- In what ways can researchers help to better address the psychological issues and distress that many people with MS face?
- In what ways does having MS impact on your psychological wellbeing?
- What are some of the main issues/ concerns that you worry about in the context of having MS?
- What sorts of coping mechanisms do you use to overcome these difficulties?
- What sorts of support systems do you use/ have available to you?

**Internet-Based Interventions**

- How regularly do you use the Internet?
- Do you have regular access to the Internet/ a computer?
- How do you feel about seeking support for some of these issues over the Internet?
- How confident are you about reading and downloading things off the Internet?
- Do you have any difficulties or reservations about using the Internet?

**Mindfulness-Based Interventions**

- Do you have any experience with mindfulness meditation or mindfulness-based intervention programs and would you be open to learning about these?
- What do you know about mindfulness meditation?

**Cognitive-Behavioural Therapy**

- Do you have any experience with cognitive behavioural therapy?
- Have you had any experience with psychological therapy to help cope with some of the problems that might arise associated with adapting to having a diagnosis of MS?
- What worked for depression/ anxiety/fatigue/pain?
- What didn’t work?

**Therapy Adherence:**

- How many sessions of internet-based therapy would you be willing/ able to commit to?
- Would you be interest in participating in a study such as this? Why/ Why not?
- What sorts of barriers do you think you would face that would prevent you from adhering to the treatment schedule?
- How viable would it be to ask you to spend 30 minutes, twice a day to work on mindfulness meditation as part of your therapy work?
- Do you have any reservations about being given work to do in between therapy module?
- Do you foresee any barriers to therapy adherence?
- DO you have any idea about how we could reduce participant drop-out?

**Therapy Preferences:**

- Would you like to be contacted over the phone/ email for technical/ therapeutic support?
- Would you be willing to come in for a follow-up session, 1 month and 3 months post-intervention to discuss what worked/ what needs remodelling?
- How interactive do you think the program needs to be? i.e. Downloading materials and doing work between session on one’s own accord vs. Downloading worksheets, filling them out, then re-uploading them on the system.
- Would case examples be helpful or irrelevant/off-putting? (i.e. *Jenny was diagnosed with MS last year.. She has been having difficulty getting tasks done at home because she has been experiencing feeling of low mood and extreme fatigue…*)
- Would you like to receive SMS/ email reminders to complete session modules?
