# Supplementary material for: Development of a Web-Based Mindfulness Program for People With Multiple Sclerosis: Qualitative Co-Design Study
Source: J Med Internet Res. 2021 Mar 2;23(3):e19309. doi: 10.2196/19309 (PMC7967236; doi:10.2196/19309)
Supplement: Multimedia Appendix 2 [file jmir_v23i3e19309_app2.pdf]

Multimedia Appendix 1. Examples of how themes derived from end-user interviews were incorporated into the program

Theme 1: Uncertainty

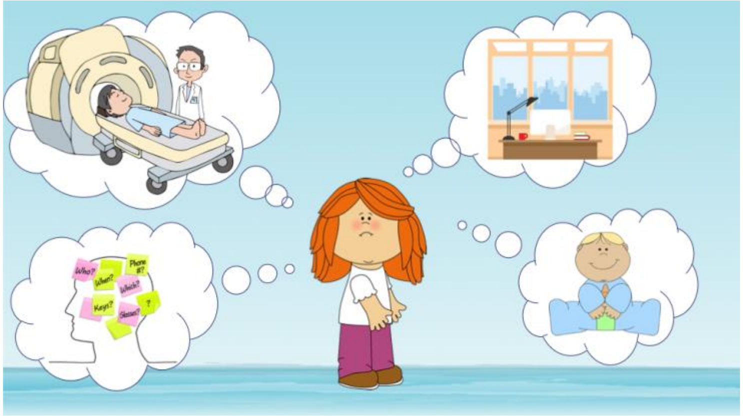

Jen used to spend much of her time worrying about things, which can be really common for people with MS: She worried about her memory, whether she'd have another relapse, whether she would be able to continue in the job that that she loved… She also worried about her children and who would look after them if she was no longer able to.

Theme 2: Grief and Loss

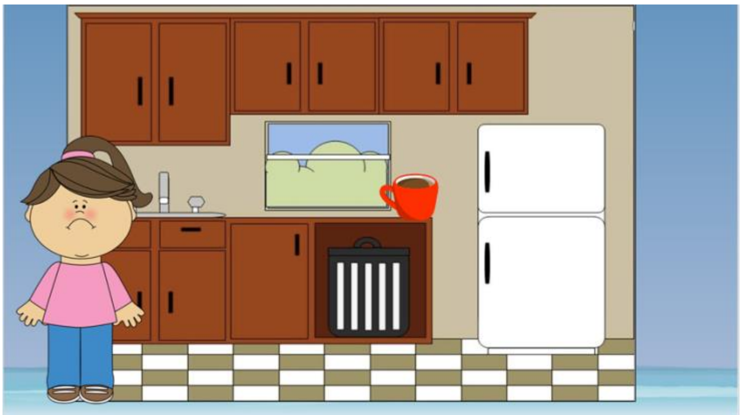

One day, Lucy was at home in the kitchen, making herself a cup of tea. She had been doing the household chores, and without thinking, she turned around to reach for the kettle too quickly, her legs, buckling beneath her. Lucy fell onto the kitchen floor. She had been having some problems with balance and muscle weakness in her legs, but she couldn't be sure whether this fall was due to her MS or her lack of concentration. In the past, she knew she would have simply laughed at a silly fall thing like this, but today, Lucy didn't feel like laughing.

Theme 3: Social isolation

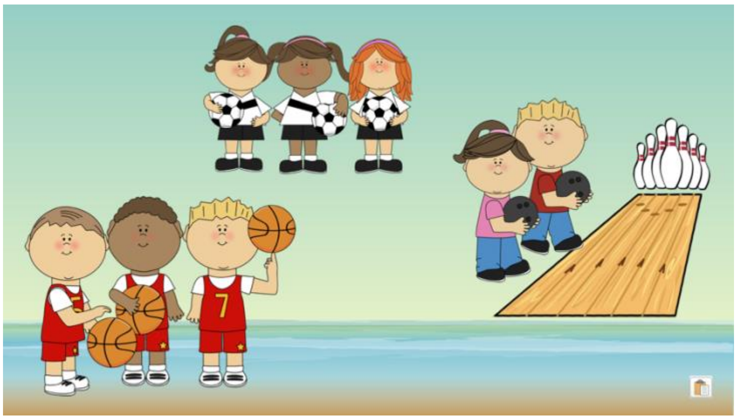

The ability to connect and receive support from others is an integral part of adjusting to life with a chronic illness like MS. Yet, many people with MS report feeling isolated and alone. Lucy, who was having problems with low mood and fatigue, found that she could no longer play social sports like she used to, and it was harder to meet up with her friends.

Character graphics by Laura Strickland/ MyCuteGraphics.com
